# Feasibility of neonatal intravenous nutrition for the management of gastroschisis in sub-Saharan Africa

**DOI:** 10.1136/wjps-2025-001133

**Published:** 2026-05-22

**Authors:** Naomi Wright, Piero Alberti, Aarinola Olaiya, Francis Abantanga, Alhassan Abdul-Mumin, Michael Amoah, William Appeadu-Mensah, Zaitun Bokhary, Bruce Bvulani, Mulenga Mulewa, Bip Nandi, Boateng Nimako, Godfrey Sama Philipo, Stephen Tabiri, Abiboye Yifieyeh, Kate Arnold, Anne Greenough, Nick Sevdalis, Andrew Leather, Sandhia Naik, Niyi Ade-Ajayi

**Affiliations:** 1 Department of Paediatric Surgery, Oxford University Hospitals NHS Foundation Trust, Oxford, UK; 2 Department of Paediatric Surgery, King’s College Hospital, London, UK; 3 Department of Surgery, Tamale Teaching Hospital, Tamale, Ghana; 4 Department of Surgery, Komfo Anokye Teaching Hospital, Kumasi, Ghana; 5 Department of Paediatric Surgery, Korle Bu Teaching Hospital, Accra, Ghana; 6 Department of Paediatric Surgery, Muhimbili National Hospital, Dar es Salaam, Tanzania; 7 Department of Paediatric Surgery, University Teaching Hospital, Lusaka, Zambia; 8 Department of Paediatric Surgery, Arthur Davison Children Hospital, Ndola, Zambia; 9 Department of Paediatric Surgery, Kamuzu Central Hospital, Lilongwe, Malawi; 10 Department of Nutrition and Dietetics, King’s College Hospital, London, UK; 11 Department of Women and Children’s Health, School of Life Sciences, Faculty of Life Science and Medicine, King’s College London, London, UK; 12 Centre for Behavioural and Implementation Science Interventions, Yong Loo Lin School of Medicine, National University of Singapore, Singapore; 13 Department of Population Health Sciences, King’s College London, London, UK; 14 Department of Paediatric Gastroenterology, The Royal London Hospital, London, UK

**Keywords:** Child Health, Child Nutrition Sciences, Health services research, Infant Nutrition Disorders

## Abstract

**Introduction:**

Parenteral nutrition (PN) is a life-saving intervention for neonates with gastroschisis. As most low and middle-income countries lack access to comprehensive neonatal PN, we evaluated the feasibility and safety of a pragmatic intravenous nutrition strategy for neonates with gastroschisis in sub-Saharan Africa.

**Methods:**

Local protocols for partial parenteral nutritional support and early enteral feeding were developed across the seven pediatric surgery centers participating in the Gastroschisis Interventional Study (*n*=192). Protocols aimed to provide up to 100 kcal/kg/day using 10% dextrose as maintenance fluid, with amino acid preparations and lipid emulsions added when locally available. Trophic breastmilk was commenced on admission, with enteral feeds advanced from the day following defect closure.

**Results:**

In the postintervention phase (*n*=127), all centers administered 10% dextrose as maintenance fluid. Six centers provided parenteral amino acid, and two centers additionally provided parenteral lipid. The proportion of neonates receiving parenteral nutritional support with 10% dextrose and amino acids increased from 18.5% to 66.1% between the pre- and postintervention phases. The median duration of parenteral nutritional support among survivors was 14 days (interquartile range (IQR): 9–22 days). Intravenous nutritional support was mainly administered by peripheral venous access, with no major clinical complications observed.

**Conclusions:**

Partial neonatal intravenous nutritional support using locally available products was feasible and safe across study centers. This approach represents a pragmatic interim strategy in settings where neonatal PN is unavailable. Expanding access to neonatal PN remains essential to achieving Sustainable Development Goal 3.2 to end preventable neonatal deaths by 2030.

**WHAT IS ALREADY KNOWN ON THIS TOPIC**

- Unacceptable disparities in mortality from gastroschisis persist between high-income and low and middle-income countries (LMICs). Neonatal parenteral nutrition (PN) is a life-saving intervention for infants with gastroschisis, yet it remains inaccessible in most of sub-Saharan Africa due to resource constraints. While recent reports suggest that ‘partial PN’ using locally available carbohydrate, protein and lipid sources may be a feasible pragmatic approach to improving outcomes, prospective multicenter evidence is limited.

**WHAT THIS STUDY ADDS**

- Across seven pediatric surgery centers in four sub-Saharan African countries, implementation of intravenous nutrition protocols based on locally available resources was feasible, with two-thirds of neonates receiving parenteral protein and protocol adherence achieved in over three quarters of cases. While nutritional support was mainly provided peripherally and with limited electrolyte monitoring, no major clinical complication was observed. Exploratory analyses indicated that provision of partial PN was associated with lower mortality.

**HOW THIS STUDY MIGHT AFFECT RESEARCH, PRACTICE OR POLICY**

- Where full neonatal PN is not consistently available, partial intravenous nutrition using locally available products is a feasible interim strategy to support neonatal surgical care. Research efforts should focus on defining the incremental impact of intravenous nutrition on outcomes, implementing scalable strategies to confirm neonatal line position in LMICs and delineating the optimal combinations of enteral and parenteral nutritional support for low-resource settings. Our findings should encourage neonatal and pediatric surgical teams in LMICs to develop local partial PN protocols and support implementation of accelerated enteral feeding protocols for neonates with gastroschisis in both LMICs and HICs. Policy recommendations must emphasize that partial PN is only a stopgap in the absence of full neonatal PN. Striving towards universal access to neonatal PN must remain a global priority to end preventable neonatal morbidity and mortality.

## Introduction

 Gastroschisis is a full-thickness congenital abdominal wall defect characterized by herniation of the gastrointestinal (GI) tract outside the abdominal cavity, affecting approximately 1 in 3000 live births.^1^ The management of gastroschisis is resource-intensive, requiring specialized neonatal intensive care, surgical expertise for bowel reduction, management of GI complications and abdominal closure, and prolonged nutritional support.^1^ Outcomes from gastroschisis are excellent in high-income countries (HICs), with mortality rates as low as 1%, while mortality is far higher in middle-income and low-income countries, with rates of 32% and 90%, respectively.^2^

In HICs, more than 90% of neonates with gastroschisis have ‘simple gastroschisis’, in which the bowel is intact and can be reduced into the abdominal cavity shortly after birth. These infants typically experience a transient postoperative ileus, which delays the establishment of enteral feeding but resolves over time.^3 4^ A minority of neonates have ‘complex gastroschisis’, which presents with bowel necrosis, perforation or atresia and typically requires repeated surgical interventions, potentially resulting in the development of short bowel syndrome, with prolonged dependence on parenteral nutrition (PN) and substantially higher morbidity and mortality.^5^ A national cohort study from the United Kingdom (UK) demonstrated that in simple gastroschisis, PN is typically required for 2 to 5 weeks (median 20 days, interquartile range (IQR): 14–34 days), whereas infants with complex disease may require PN for several months.^6^,^8^

The delayed return of bowel function after closure of the abdominal defect in neonates with gastroschisis is thought to result from prolonged exposure of the bowel to amniotic fluid in utero, handling during surgical reduction and increased intra-abdominal pressures after reduction.^3 4^ During this period, administration of PN on the neonatal intensive care unit (NICU) is a life-saving intervention that provides calories and essential substrates to mitigate catabolism and support nitrogen balance, maintaining tissue integrity and immune function until enteral feeding is tolerated.^9^,^11^ In HIC settings, nutritional support is typically delivered using standardized, industrially manufactured neonatal PN formulations providing a full complement of macronutrients, micronutrients and electrolytes.^12^

The importance of nutritional support is particularly pronounced in LMIC settings, where delayed presentation and inadequate prehospital resuscitation increase the likelihood of neonates presenting with dehydration, hypothermia and sepsis—key drivers of mortality in gastroschisis.^2 13 14^ Comprehensive neonatal PN, however, remains largely inaccessible in most LMICs due to limited availability of the key resources and infrastructure required for safe and sustained administration. These include standardized premade neonatal PN formulations, in-hospital pharmacy and laboratory services for admixture and electrolyte monitoring, specialized neonatal gastroenterology and pharmacy support, availability of central venous access devices, regularly audited infection prevention protocols and access to mobile radiography to confirm the position of lines after insertion.^215^,^17^ Additional challenges include the unreliability of PN supply chains in low-resource contexts and the short shelf life of neonatal PN formulations when available.^10^ To our knowledge, South Africa is currently the only country in sub-Saharan Africa where standardized neonatal PN solutions are locally manufactured and distributed.^2 15 18 19^

In response to these constraints, a growing body of evidence from LMIC centers suggests that ‘partial PN’ strategies combining intravenous glucose (dextrose) with amino acid (AA), lipid and/or multivitamin preparations may offer a feasible and potentially life-saving alternative for neonates with surgical conditions, including abdominal wall defects and intestinal atresia.^20^,^22^ We report our experience in developing and implementing intravenous nutrition protocols for neonates with gastroschisis across the seven centers participating in the Gastroschisis Interventional Study (2018–2021).^15^

## Methods

The following guidelines were complied with to optimize the design, evaluation, and results reporting for the Gastroschisis Interventional Study^23^,^29^: Standard Protocol Items: Recommendations for Interventional Trials (SPIRIT), Medical Research Council guidance on developing and evaluating complex interventions, Standards for reporting implementation studies (StaRI), Template for Intervention Description and Replication (TIDieR), Standards for Quality Improvement Reporting Excellence (SQUIRE).

Seven pediatric surgery centers in Ghana (*n*=3), Zambia (*n*=2), Malawi (*n*=1) and Tanzania (*n*=1) participated in the study.^15^ During the exploration phase, a systematic literature review and qualitative study were undertaken to identify the core and adaptable components of the interventional bundle.^15 19 30^ This was followed by a consensus process to develop provisional center-specific care bundles. In the early stages, over two-thirds of multidisciplinary team (MDT) members were in favor of attempting to provide full neonatal PN. Discussions with MDT members in each center, however, concluded that attempting to source neonatal PN from South Africa would be prohibitively expensive and compromise the sustainability of the bundle.

With support from a UK-based pediatric gastroenterologist specialized in neonatal PN and advanced enteral feeding strategies, local gastroenterologists liaised with pharmacists in each center to identify available resources to incorporate into protocols to partially support nutrition. At the start of the 4-week implementation phase, the study lead met face-to-face with MDT members in each center, including neonatologists, pediatricians, pediatric surgeons, nurses and pharmacists, to finalize center-specific protocols that were then circulated electronically. These covered care in the referring district hospitals, resuscitation and cotside management of the gastroschisis in the study center, accelerated breastfeeding and intravenous nutrition.^19^ During the implementation phase, a pediatric gastroenterologist and neonatal nurse specialist attended each study center for two weeks to support the local MDT in sourcing, preparation, storage and administration of intravenous nutritional support.^19^

Across all study centers, nutrition protocols recommended the use of 10% dextrose as maintenance fluid to support energy provision. All centers planned to commence intravenous nutritional support on admission, building up to full volume over the first 4 days of life. Six centers included intravenous AA preparations in their nutrition protocols. In Ghana, Korle-Bu Teaching Hospital (KBTH) used Astymin 3 (Tablets India; online supplemental appendix 1), while Komfo Anokye Teaching Hospital (KATH) used Celemin 10 (Otsuka Pharmaceuticals India Pvt; online supplemental appendix 2). Tamale Teaching Hospital (TTH) used Celemin 10 at the beginning of the intervention phase but later switched to Astymin 3 due to local availability. Additionally, KATH was able to incorporate Celepid (Claris Lifesciences) lipid emulsion into its intravenous nutrition protocol, while KBTH provided micronutrient supplementation by adding intravenous multivitamin Pabrinex (Kyowa Kirin International) to its protocol. In Zambia, University Teaching Hospital Lusaka (UTHL) and Arthur Davison Children’s Hospital (ADCH) used Astymin 3. In Tanzania, Muhimbili National Hospital (MNH) used Kabiven Peripheral (Fresenius Kabi AG), a standardized formulation of adult PN modified for peripheral use, which contains dextrose, Vamin 18 AA solution, Intralipid emulsion and electrolytes^31^ (online supplemental appendix 3). There were no available intravenous macronutrient preparations in Malawi. While the study team managed to arrange delivery of Astymin 3 from Zambia, the local MDT at Kamuzu Central Hospital (KCH) decided that administration would not be safe and sustainable in their setting and therefore did not implement an intravenous nutrition protocol, focusing instead on caloric support with 10% dextrose and early advancement of enteral feeds. Two centers, KBTH (Ghana) and MNH (Tanzania), were able to autonomously source intravenous macronutrients in advance of the implementation phase and started administering these during the preintervention phase.

Electronic and paper versions of the local intravenous nutrition protocol were distributed to the medical, nursing, dietetics, and pharmacy staff responsible for administration and monitoring on the neonatal unit. Daily carbohydrate and protein provisions, as well as lipid where available, were calculated and adjusted by neonatal intensive care nurses according to infants’ weight and day of life. Protocols specified the intended route of administration as well as electrolyte and metabolic monitoring intervals where available. By the fourth day of life, intravenous nutrition protocols including carbohydrates, AAs and lipids were designed to meet over 80% of neonates’ daily calorie requirements (table 1).

**Table 1:** Build-up of intravenous nutrition for neonates with gastroschisis (example of a study protocol including parenteral lipid)

Day of life	Glucose (g/kg/day) (mL/kg/day)	Protein (g/kg/day) (mL/kg/day)	Lipid (g/kg/day) (mL/kg/day)	Total fluid intake* (mL/kg/day)	Non-protein calories (kcal/kg/day)	Total calories (kcal/kg/day)
First	5 (50)	1 (10)	1 (9)	60	29	33
Second	7 (70)	2 (20)	1.5 (13.5)	90	41.5	49.5
Third	9 (90)	3 (30)	2 (18)	120	54	66
Fourth onward	12 (120)	3 (30)	2.5 (22.5)	150	70.5	82.5

* Total fluid intake (TFI) includes dextrose and amino acid volumes. Parenteral lipid, where available, was administered separately in addition to the specified TFI.

The interventional bundle in each center included a protocol for early advancement of enteral feeds. Across centers, enteral feeding protocols included placing of a nasogastric (NG) tube on free drainage with 2-hourly to 4-hourly aspirates, replacing NG losses with intravenous crystalloid fluids (0.9% NaCl with potassium or lactated Ringer’s) in addition to maintenance fluids, and providing trophic expressed breastmilk (EBM; 1 mL 8-hourly) from admission to stimulate bowel function and provide antibodies. Some centers also provided EBM lollipops or colostrum-soaked swabs in between trophic feeds for comfort and maternal sensory exposure. Enteral feeding (either breastfeeding or EBM via NG tube) was advanced from 24 hours after defect closure, progressively scaling back intravenous nutrition and aiming to reach full enteral feeding within a week of closure. The duration and frequency of enteral feeds varied between centers in accordance with local NICU protocols. An example enteral feeding protocol is detailed in online supplemental appendix 4.

Exploratory unadjusted comparisons of mortality by macronutrient exposure were performed using Fisher’s exact test. As detailed in the published study protocol, ethical approval was gained at all participating sites. Written consent was required from the guardian holding parental responsibility for all patients included in the study.^15^

## Results

### Feasibility of protocol implementation

The Gastroschisis Interventional Study (2018–2021) included 192 patients with simple gastroschisis: 65 in the preintervention phase and 127 in the postintervention phase. The median time from arrival at the study center to initiation of fluids was 1 hour (IQR: 1 to 2 hours). The overall uptake of nutritional support across the preintervention and postintervention phases of the study stratified by macronutrient type is detailed in table 2. The proportion of patients in the postintervention phase who received parenteral nutritional support as specified in their local protocol is shown in table 3.

**Table 2:** Overall uptake of individual macronutrients before and after implementation of intravenous nutrition protocols across the seven study centers

Center	Patients		Carbohydrates (dextrose)		Amino acids		Lipids		Total
	Pre	Post	Pre	Post	Pre*	Post	Pre^*^	Post	
TTH (Ghana)	4	8	4	8	0	8	0	0	**12**
KBTH (Ghana)	9	27	8	27	8	24	0	0	**36**
KATH (Ghana)	5	19	4	18	0	19	0	11	**24**
UTHL (Zambia)	7	13	7	13	0	7	0	0	**20**
ADCH (Zambia)	2	14	2	14	0	10	0	0	**16**
KCH (Malawi)	25	30	25	29	0	0	0	0	**55**
MNH (Tanzania)	13	16	7	16	4	16	4	6	**29**
Total	**65**	**127**	**57**	**125**	**12**	**84**	**4**	**17**	**192**
% of patients	**100%**	**100%**	**87.7%**	**98.4%**	**18.5%**	**66.1%**	**6.2%**	**13.4%**	

* KBTH (Ghana) and MNH (Tanzania) were able to locally source and started to administer intravenous macronutrients in the preintervention phase

ADCH, Arthur Davison Children’s Hospital; KATH, Komfo Anokye Teaching Hospital; KBTH, Korle-Bu Teaching Hospital; KCH, Kamuzu Central Hospital; MNH, Muhimbili National Hospital; TTH, Tamale Teaching Hospital; UTHL, University Teaching Hospital Lusaka.

**Table 3:** Per-protocol intravenous macronutrient uptake in the postintervention phase

Center	Carbohydrates (dextrose)	Amino acids	Lipids	Total
TTH (Ghana)	8/8 (100%)	8/8 (100%)	N/A	**8/8** (**100%**)
KBTH (Ghana)	27/27 (100%)	24/27 (88.8%)	N/A	**24/27** (**88.8%**)
KATH (Ghana)	18/19 (94.7%)	19/19 (100%)	11/19 (57.9%)	**11/19** (**57.9%**)
UTHL (Zambia)	13/13 (100%)	7/13 (53.8%)	N/A	**7/13** (**53.8%**)
ADCH (Zambia)	14/14 (100%)	10/14 (71.4%)	N/A	**10/14** (**71.4%**)
MNH (Tanzania)	16/16 (100%)	16/16 (100%)	6/16 (37.5%)	**6/16** (**50%**)
**Total**	**96/97** (**99.0%**)	**84/97** (**86.6%**)	**17/35** (**48.6%**)	**66/97** (**68.0%**)

Percentages refer to the proportion of neonates in the postintervention phase who received all macronutrients recommended by their local protocol. Cells marked N/A indicate macronutrients not included in local protocols and therefore excluded from feasibility analyses. KCH (Malawi) is not included as it did not provide parenteral nutritional support with intravenous AAs and/or lipid.

ADCH, Arthur Davison Children’s Hospital; KATH, Komfo Anokye Teaching Hospital; KBTH, Korle-Bu Teaching Hospital; MNH, Muhimbili National Hospital; TTH, Tamale Teaching Hospital; UTHL, University Teaching Hospital Lusaka.

As complete neonatal PN was not included under any center-specific protocol, all neonates received energy provision and substrate supplementation to different extents, but none received comprehensive nutritional support.

All centers provided intravenous caloric support, with 99.0% of neonates in the postintervention phase receiving intravenous 10% dextrose. Except for KCH (Malawi), all centers provided nutritional support with AA preparations. The overall proportion of patients receiving parenteral AA increased from 18.5% to 66.1% between the preintervention and postintervention phase. Only two centers were able to include parenteral lipid in their local protocol. As a proportion of the overall study population in each phase, uptake of lipid increased from 6.2% to 13.4% between the preintervention and postintervention phase. Across the six centers that provided parenteral nutritional supplementation, 68.0% of neonates received the full complement of macronutrients specified in their local protocol (table 3).

Among patients who received nutritional support and survived to discharge in the postintervention phase, the median duration of intravenous nutritional support was 14 days (IQR: 9–22 days). Among survivors in the postintervention phase, enteral feeds were started at a median of 6 days after defect closure (IQR: 3–8 days) and progressed to full feeds at a median of 14 days following closure (IQR: 8–18 days). Nearly all patients (42/44) received breast milk only, with two patients receiving both breast milk and formula milk.

### Macronutrient delivery and resource implications

#### Carbohydrates

Intravenous carbohydrate in the form of dextrose solution is widely used as neonatal maintenance fluid and low-density (4 kcal/g) source of non-protein calories. Across all centers, study protocols specified 10% dextrose solution (D10W) as the maintenance fluid of choice. This provided the highest caloric support achievable with peripheral administration, as higher concentrations would require central venous access due to the risk of thrombophlebitis.^32^ While lower concentrations (*e.g.*, 4.3% or 5%) were used in the preintervention phase, all centers transitioned to 10% dextrose postintervention. A total of 179 patients (93.2%) across both phases received dextrose, with the remainder receiving Darrow’s solution, lactated Ringer’s or 0.9% sodium chloride. Sodium and potassium supplementation was added to maintenance fluids according to the local NICU protocols. Build-up of 10% dextrose enabled almost two-thirds of patients’ 100 kcal/kg/day calorie requirement to be met by the fourth day of life (table 1).

#### Protein

In neonates unable to feed enterally, parenteral AAs are essential to limit early postnatal catabolism. The AA solutions Astymin 3 and Celemin 10 contain essential AAs and provide the same caloric yield (4 kcal/g) as 10% dextrose. They were included in protocols for six out of seven centers, reflecting good availability in the LMIC context and limited cost (1$/patient/day). When affordability challenges arose, bottles could be shared between patients using sterile precautions and the cost divided among families.^20^ By contrast, Kabiven Peripheral cost around $10/patient/day in the center that administered it (MNH, Tanzania). Challenges with inconsistent availability limited implementation in Zambia, leading to postintervention administration rates of 53.8% in UTHL and 71.4% in ADCH. Similarly, AA preparations were not included in the protocol for KCH (Malawi) due to local unavailability. Given the fixed fluid constraints of the first week of life, study protocols specified that aqueous AA preparations should be administered by substituting an equivalent volume of 10% dextrose to an unaltered total fluid intake and coinfused as a single AA-dextrose maintenance mixture by the same peripheral venous line (online supplemental appendix 1-3). Overall, 86.6% (84/97) of neonates in the postintervention phase received intravenous AAs when this was included in their local study protocol (table 3).

#### Lipids

Parenteral lipid emulsions are a dense (9 kcal/g) source of non-protein energy, supply essential fatty acids and are necessary for the absorption of fat-soluble vitamins A, D, E and K.^33^ Lipid emulsions like Celepid and Intralipid are available in 10% (1 kcal/mL) and 20% (2 kcal/mL) formulations (online supplemental appendices 2–3). Among protocol components, limited and inconsistent availability of lipid emulsions posed the greatest implementation challenge. Only KATH (Ghana) and MNH (Tanzania) were able to include parenteral lipid in their protocol. In these two centers, lipid availability was limited by procurement constraints and the need to distribute available supplies across both neonatal and older pediatric patients requiring nutritional support. Consistent delivery of parenteral lipid in the postintervention phase was only achieved for 17 neonates, corresponding to 48.6% of eligible patients (table 3). Study protocols specified that intravenous lipid should be infused continuously over 24 hours in addition to maintenance fluid, administered via a dedicated intravenous line using aseptic non-touch technique, stored refrigerated between infusions and protected from light during administration to minimize photo-oxidative degradation.

### Supplements, monitoring and vascular access

A key consideration during the development of intravenous nutrition protocols concerned the prevention of electrolyte imbalance and micronutrient deficiency. In our study, only KBTH (Ghana) could provide limited micronutrient supplementation to neonates in the postintervention phase by adding Pabrinex to its protocol. MNH (Tanzania) was the only center where routine electrolyte monitoring on NICU was available, which allowed for comprehensive electrolyte supplementation through short-term administration of Kabiven Peripheral (online supplemental appendix 3). In other centers, sodium (2–3 mmol/kg/day) and potassium (1 mmol/kg/day) supplementation was added to maintenance dextrose based on the results of monitoring when available, clinical status and NG fluid losses. Despite the lack of intermittent availability of biochemical monitoring, no clinical evidence of either hypernatremia or hyponatremia, hyper- or hypokalemia, or other electrolyte imbalances was observed.

All centers used peripheral cannula for intravenous nutrition. Four centers (TTH, Ghana; KATH, Ghana; UTHL, Zambia; MNH, Tanzania) included neonatal (2 Fr) peripherally inserted central catheters (PICCs) in their gastroschisis protocol. NICU and pediatric surgical team members were trained in PICC insertion and line care using simulation models. Three centers decided against PICC use due to safety and infection concerns. One center (KCH, Malawi) introduced umbilical vein catheters (UVC) for initial resuscitation but not nutrition. Across both phases, 28 patients (14.6%) received a PICC and 3 patients (1.6%) received a UVC, with all others receiving peripheral cannulae. Over half (58.1%) of PICCs and UVCs were inserted on the day of admission.

Several difficulties relating to intravenous access were noted. Peripheral intravenous access was most feasible but frequently required repeat reinsertions due to the short lifespan of cannulae. This could result in prolonged periods of time without intravenous access with the risk of patient deterioration due to the lack of hydration, nutrition and antibiotic delivery. Nursing concerns around line care and infection risk limited the use of UVCs. Despite their availability, the lack of portable X-ray to confirm line position constrained the use of neonatal PICCs. Transporting patients to the radiology department would increase the risk of deterioration in a setting without adequate resources, negating the rationale of a care protocol that emphasized cotside silo application, bowel reduction and closure to avoid the risks of transporting unstable neonates to the operating theater. Other difficulties with PICC use included the time taken for insertion for busy clinicians and difficulty with maintaining patency, line position and infection control. All centers eventually relied on peripheral cannulae as their main route for intravenous nutritional support.

Three patients had a complication due to central access, including one PICC-related sepsis, one PICC extravasation injury and one case of lower limb edema due to UVC malposition. No complications due to peripheral delivery of intravenous nutrition (*e.g.*, extravasation-associated necrosis or limb loss) were reported.

### Exploratory outcome analysis

In exploratory unadjusted analyses of the postintervention cohort, mortality differed by receipt of parenteral macronutrient supplementation. Neonates who received parenteral AAs had lower mortality than those who did not (55.4% (46/83) *vs.* 79.5% (35/44); RR=0.70, 95% CI 0.55 to 0.89; Fisher’s exact *p*=0.01). Mortality was also lower, although not significantly, among neonates who received parenteral lipid in addition to AAs compared with AAs alone (35.3% (6/17) *vs.* 60.6% (40/66); RR=0.58, 95% CI 0.30 to 1.13; Fisher’s exact *p*=0.09).

## Discussion

For neonates with gastroschisis in sub-Saharan Africa, affordable, locally sourced carbohydrate, protein and lipid provided partial parenteral nutritional support. While not a substitute for full neonatal PN, this strategy provided a pragmatic solution: augmentation of maintenance intravenous fluids with macronutrients in settings without the infrastructure for consistent administration and monitoring of comprehensive neonatal PN.

Sub-Saharan Africa accounts for 60% of global under-5 deaths (2.8 to 3.3 million annually) and has the highest neonatal mortality worldwide at 27 deaths per 1000 live births.^34^,^36^ Around 30% of 13.4 million annual preterm births occur in sub-Saharan Africa.^37 38^ Prematurity is the leading cause of under-5 and neonatal mortality globally, with around half of very premature neonates in LMICs dying from lack of key interventions including nutritional support.^37 38^ Prematurity and its complications, such as necrotizing enterocolitis, are the most common indications for neonatal PN.^39^ UK guidelines recommend initiating PN in all neonates born at or before 31 weeks (or 1.250 kg) and in those unable to establish sufficient enteral feeding in the first 48–72 hours of life.^40^ Other indications include sepsis, congenital heart disease, GI infections, inborn errors of metabolism and surgical diseases such as gastroschisis or intestinal atresia.^39 41^ Most neonates require PN for less than a month, and live healthy lives thereafter.^42^ Failure to provide neonatal PN in low-resource settings is therefore associated with substantial mortality, morbidity and loss of disability-adjusted life years.^43^

### Feasibility and efficacy

Across all centers, 66.1% of neonates in the postintervention phase of the Gastroschisis Interventional Study received partial PN by addition of AAs to dextrose, with 13.4% also receiving lipid. Protocols for administrations of intravenous macronutrients were developed for six out of seven participating centers. Augmentation of maintenance carbohydrates with parenteral protein was highly feasible, with 86.6% of neonates in these centers receiving intravenous AAs in the postintervention phase. Lipid emulsions were incorporated into nutrition protocols by two centers. Availability challenges significantly constrained protocol implementation, with only 48.6% of neonates in the two centers receiving parenteral lipid in the postintervention phase. Given the key role of parenteral lipid as a dense source of calories that preserves endogenous protein stores when enteral feeding is delayed, research efforts should focus on identifying strategies to improve access to lipid, evaluate local barriers to administration and investigate its incremental impact on neonatal surgical outcomes. Following study completion, participating centers continued to adapt and refine nutrition protocols in response to local clinical needs and now routinely administer combinations of dextrose, AAs and lipids to neonates with gastroschisis, with some extending these approaches to other surgical and non-surgical neonates requiring nutritional support.

A preliminary analysis of study outcomes observed a reduction in mortality from 80% to 65% between study phases.^19^ Receipt of parenteral nutritional support was associated with higher survival in the postintervention phase. Mortality was approximately 30% lower in neonates who received protein than those who did not, with a trend towards 40% lower mortality in patients who also received lipid. Without multivariable analysis, it is not possible to determine whether this association reflects a mortality benefit or survivorship bias, as neonates had to survive long enough to receive nutritional support. Insights can be drawn from KCH (Malawi), where parenteral protein was not available and neonates only received dextrose. KCH did not achieve a reduction in mortality between study phases (80% *vs.* 83.3%,). The clinical course of neonates in KCH suggested a plausible link between absence of parenteral protein and poor outcomes. Over the first 2 weeks of life, neonates in KCH developed progressive edema due to depletion of endogenous nitrogen stores. Unless sufficient feeding was established, catabolic deterioration resulted in anasarca and death by malnutrition. Since the end of the study, however, ongoing implementation of the care bundle at KCH was associated with a reduction in mortality to 71.7% despite continued absence of PN, with 85.9% of deaths occurring in the first 10 days of life.^44^ This suggests that survival from gastroschisis in LMICs may be most dependent on effective resuscitation and treatment of hypovolemia, electrolyte imbalance and sepsis, with parenteral nutritional support providing a later survival benefit in neonates with a delay in establishing feeding.

### Safety considerations

Safety concerns during study design and implementation were primarily related to difficulties in securing reliable central venous access and limited monitoring capacity. In HICs, neonatal PN is delivered via central venous access using electrolyte-rich, hyperosmolar formulations that require regular monitoring and pose a high risk of extravasation injury if given peripherally. Despite limited monitoring capacity and reliance on peripheral access, our study observed no clinical evidence of metabolic disturbance, electrolyte imbalance or extravasation injury. These findings must be interpreted in the context of the deliberately dilute and incomplete nutritional regimens implemented and do not exclude a higher risk of complications with more comprehensive formulations. Limited monitoring in most study centers further constrains inferences regarding safety, as the development of asymptomatic iatrogenic abnormalities cannot be excluded.

In MNH (Tanzania), modified adult PN (Kabiven Peripheral) was used pragmatically. While short-term use appeared safe, adult PN is not suited to neonatal nutrient requirements and renal electrolyte handling and cannot be recommended in LMIC units without regular monitoring capacity. In keeping with this, after completion of the study, MNH transitioned away from use of Kabiven Peripheral in favor of a focus on earlier advancement of enteral feeding. Limited PN availability and monitoring capacity were cited as major barriers to intravenous nutritional support in their setting.

Reliability of vascular access is a critical safety challenge for neonatal care in LMICs. Studies in MICs report that use of conventional central venous catheters is often associated with sepsis and non-infective complications, including arrhythmias and pleural effusions.^45 46^ While peripheral cannulas are frequently used for nutrition in LMICs, evidence on the safety and efficacy of peripheral PN is limited due to concerns about extravasation injury.^15 47^ In their review of neonatal PN use in South Africa, Murguia-Peniche and Kirsten recommend that, if PN is given peripherally, insertion sites should be monitored hourly and the cannula changed every 72 hours.^48^

PICCs and UVCs have been described in both HICs and LMICs as preferred routes of access for neonatal nutritional support, allowing central venous administration with fewer cannulation attempts.^49^ In LMICs, the Global PaedSurg study found that PICC use was associated with reduced mortality from GI congenital anomalies.^2^ However, the use of PICCs in the LMIC context has been shown to be associated with a substantial risk of complications including infection, occlusion and malposition.^50^ UVCs are particularly useful for resuscitation of gastroschisis neonates, who are typically maximally vasoconstricted at presentation.^51 52^ In India, a trial of neonatal PICCs and UVCs found that these did not differ in success rate, insertion time or complication rate, although UVCs were cheaper and had a shorter mean duration of use.^53^ Retrospective studies have shown no difference in infection risk between PICCs and UVCs.^54^

While our study successfully implemented intravenous nutrition protocols, vascular access challenges limited delivery. As interruptions in access were common and macronutrient availability was often inconsistent, most neonates likely received a lower cumulative nutrient volume than indicated by their recorded duration of partial PN. Without portable radiography, most PICCs remained unused despite training, and local teams relied on peripheral cannulae as the primary route of intravenous access. Point-of-care ultrasound (POCUS) may represent a scalable solution to support neonatal line positioning in LMICs, given its portability and multipurpose use. POCUS has increasingly been evaluated as an alternative to radiography for neonatal PICC tip localization, with studies in HIC NICUs showing high sensitivities even for very small-caliber catheters (1–2 Fr) and reduced need for repositioning and reimaging.^55^,^58^ However, utility remains limited by high operator dependency. The key implementation question in LMICs is whether POCUS-guided approaches can be implemented with sufficient reliability, given variable equipment quality and training capacity, to permit safe confirmation of line position without portable radiography fallback. These challenges highlight the need for comparative multicenter research to define more comprehensive neonatal nutritional strategies suitable for peripheral administration and to establish scalable and cost-effective approaches to neonatal central venous access in LMICs, with sufficient granularity in data collection to capture interruptions in vascular access and supply, so that cumulative macronutrient delivery can be accurately quantified at the patient level.

### Neonatal nutritional support in LMICs

The absence of comprehensive neonatal PN has led neonatology and pediatric surgery teams in LMICs to develop surrogate strategies for nutritional support.^19^ In gastroschisis, accelerated enteral feeding protocols have improved survival, with reported mortalities of 58% (Kenya), 67% (Uganda) and 84% (Zimbabwe).^59^,^61^ In Kenya, Kuremu *et al* found that patients could tolerate full enteral feeds advanced from the first week of life by 7 days after closure.^59^ In Uganda, Wesonga and Situma infused 10% dextrose while teaching mothers to give neonates gauze soaked in breast milk. Once stool was passed, the NG tube was clamped and breastfeeds advanced, reducing median time to full feeds to 14 days (IQR: 7–21 days).^60^

Use of intravenous AAs and lipids has been shown to be a feasible and cost-effective strategy to improve survival in gastroschisis.^5962^,^64^ Studies in Nigeria and Uganda described improved outcomes after addition of AA infusions alongside dextrose and multivitamins.^21 60^ In Kenya, Kuremu *et al* described improved survival after introducing intravenous albumin and AA in their gastroschisis bundle.^59^ In Jamaica, Marshall Niles *et al* found that neonates receiving partial PN with peripheral dextrose and AA had an almost 30% lower mortality than those receiving no nutritional support.^62^

Evidence on use of full neonatal PN in LMICs is limited by resource constraints. As no centers outside South Africa have access to premanufactured neonatal PN, adult PN is typically used with resultant wastage and dosing errors.^19^ Even when PN, gastroenterologists and nutrition nurses are available, PN deployment may be limited in LMIC centers by cost implications for families and laboratory monitoring constraints.^65^ Nonetheless, case series from Malaysia, Mexico and Afghanistan have suggested that even short durations of PN (7 to 14 days) can improve mortality to below 20%, approaching HIC figures.^66^,^68^ Strategies to expand access to neonatal PN in LMICs cannot be extrapolated from neonatal PN solutions in HICs as the concentrations, dosing regimens and monitoring schedules that underpin their safe use are incompatible with the procurement, storage, vascular access and monitoring challenges of LMICs. The optimal, practical and deliverable combination of macronutrients, micronutrients and electrolytes required to improve neonatal outcomes in LMICs is poorly defined and should be the subject of future multicenter comparative research efforts.

In conclusion, substitution of conventional neonatal PN with locally available carbohydrate, protein and lipid sources proved to be a safe and feasible strategy for providing nutritional support to neonates with gastroschisis in sub-Saharan Africa. Short-term, partial intravenous nutrition can act as a bridge to support neonates unable to tolerate enteral feeding in countries where formal neonatal PN is unavailable, with the potential to improve clinical outcomes and mortality. Neonatal surgery teams in LMICs should be encouraged to explore the feasibility of such novel intravenous nutrition regimens within locally appropriate protocols.

Ensuring that PN is available when required in LMICs remains imperative given its role as a life-saving intervention. At the same time, our findings suggest that shorter durations of peripheral intravenous nutrition may be associated with favorable outcomes in neonates requiring nutritional support when advanced enteral feeding strategies are in place. This warrants further investigation through randomized controlled trials and may have clinical implications for both LMICs and HICs, where a more conservative approach to neonatal PN and earlier transition to enteral feeding may reduce the risks and costs associated with prolonged use of conventional, centrally delivered PN.

While striving towards universal availability of PN must remain a global priority to achieve Sustainable Development Goal 3.2 to end preventable neonatal deaths by 2030, our study suggests that availability need not equate to routine and prolonged use.^69^ In low and high-resource settings, a more selective and context-sensitive approach to PN may represent an important advance to the delivery of neonatal care.

## Supplementary material

10.1136/wjps-2025-001133 online supplemental file 1

## Data Availability

All data relevant to the study are included in the article or uploaded as supplementary information.
