# Supplementary material for: Feasibility of neonatal intravenous nutrition for the management of gastroschisis in sub-Saharan Africa
Source: World J Pediatr Surg. 2026 May 22;9(3):e001133. doi: 10.1136/wjps-2025-001133 (PMC13202163; doi:10.1136/wjps-2025-001133)
Supplement: online supplemental file 1 [file wjps-9-3-s001.pdf]

APPENDICES

Appendix 1: Example of a partial neonatal parenteral nutrition protocol using Astymin 3®

Appendix 2: Example of a partial neonatal parenteral nutrition protocol using Celemin 10® with or without Celepid®

Appendix 3: Example of a neonatal parenteral nutrition protocol using modified adult PN (Kabiven Peripheral®)

Appendix 4: Example of an accelerated breastfeeding protocol used in study centres

ASTYMIN PROTOCOL FOR GASTROSCHISIS STUDY

The most suitable product is Astymin 3:

ASTYMIN 3, 200ml bottle = 1Omls contains 0.8g protein

Day of arrival to TTH	Volume of Astymin
Day 1	1g/kg protein (12mls/kg)
Day 2	2g/kg protein (24mls/kg)
Day 3 onwards	3g/kg protein (36mls/kg)

*Maintenance fluid volumes as per NICU protocol

HOW TO ADMINISTER ASTYMIN

1. Calculate the total maintenance fluid requirement for a 24 hour period.

For example, 2kg term baby on day 5 of life = 150ml/kg/day = 300mls in 24 hours.

2. Calculate volume of Astymin according to weight as above.

For example 2kg x 36mls = 72mls.

3. Take away the Astymin volume from the total maintenance fluid requirement.

For example, for a 2kg baby, 300mls – 72mls = 228mls.

4. Add the Astymin to the appropriate maintenance fluids (5% or 10% dextrose)

For example, 72mls Astymin + 228mls dextrose = 300mls.

Infuse over 24 hours (300/24 = 12.5mls/hour).

Use an aseptic non touch technique throughout. Keep the Astymin in the fridge.

GASTROSCHISIS PATIENTS

Commence Astymin on day 1 of presentation or as soon as possible.

24 hours following closure of the gastroschisis defect, breastfeeding is to start as per the breastfeeding protocol.

When breastfeeding is established at 10 minutes 6 hourly, the Astymin can be reduced by half (18mls/kg). When breastfeeding for 15 minutes 3 hourly, the Astymin can be stopped.

PARTIAL PARENTERAL NUTRITION PROTOCOL FOR GASTROSCHISIS STUDY

Komfo Anokye Teaching Hospital

AMINO ACIDS

CELEMIN 10 plus (10%), 500ml bottle 1Omls contains - 1g protein

(A suitable alternative is Astymin 3, 200ml bottle)

Day of life	Total maintenance fluid volume for 24 hours*	Volume of amino acids
1	60mls/kg	1g/kg protein (= 10mls/kg)
2	90mls/kg	2g/kg protein (= 20mls/kg)
3	120mls/kg	3g/kg protein (= 30mls/kg)
**4 and above**	**150mls/kg**	**3g/kg protein (= 30mls/kg)**

*adjust if preterm

HOW TO CALCULATE AND ADMINISTER CELEMIN

1. Calculate the total maintenance fluid requirement for a 24 hour period.

For example, 2kg baby on day 4 of life = 150ml/kg/day = 300mls in 24 hours.

2. Calculate volume of Celemin according to weight as above.

For example 2kg x 30mls = 60mls.

3. Subtract the Celemin volume from the total maintenance fluid requirement.

Example: for a 2kg baby, 300mls – 60mls = 240mls.

To make up 240mls of maintenance fluids withdraw 10mls from a 250ml bag of 10% dextrose/ 0.18% saline.

4. Add the Celemin to the dextrose/ saline.

Example : add 60mls of Celemin to 240mls dextrose/ saline = 300mls.

5. Infuse over 24 hours

Example 300mls/24hours = 12.5mls/hour.

Use Aseptic Non-Touch Technique (ANTT) throughout.

Keep the Celemin in the fridge.

LIPIDS

The lipid volume will not form part of the maintenance fluid volume – it is in addition

Celipid 10%, 500ml bottle contains 1100 kcal/litre = 1.1kcal/ml

For the purposes of this study we will round down to 1kcal/ml for calculations

Day of life	Fat given and calories from fat*	Volume of Celipid 10%
1	1g/kg (= 9kcal/kg)	9mls/kg
2	1.5g/kg (= 13.5kcal/kg)	13.5mls/kg
3	2g/kg (= 18kcal/kg)	18mls/kg
**4 and above**	**2.5g/kg (= 22.5kcal/kg)**	**22.5mls/kg**

*1g/kg is equivalent to 9kcal/kg

1. Do not mix Celipid with anything else
2. Withdraw required amount into syringe driver
3. Refrigerate bottle between infusions
4. Celipid must be given centrally
5. Infuse lipids over 24 hours
6. Use Aseptic Non-Touch Technique (ANTT)
7. Keep covered and protect from light to prevent oxidation
8. Do not leave bottle hanging

Example

2kg baby on day 4 will receive 2 x 22.5 mls Celipid = 45mls over 24 hours at 1.9 mls/hr

GASTROSCHISIS PATIENTS

Commence Celemin a on day 1 of presentation or as soon as possible.

After PICC line insertion can start Celipid

24 hours following closure of the gastroschisis defect, breastfeeding is to start as per the breastfeeding protocol.

When breastfeeding is established at 10 minutes 6 hourly, the Celemin can be reduced by half and continue with Celipid

When breastfeeding for 15 minutes 3 hourly, the Celemin and Celipid can be stopped.

PARENTERAL NUTRTION PROTOCOL FOR MUHIMBILI NATIONAL HOSPITAL

GASTROSCHISIS STUDY

The Parenteral Nutrition currently available is Kabiven peripheral Emulsion for Infusion.

This has 3 separate chambers:

1) Glucose 11%. Volume: 885 mls.

2) Vamin 18 Novum is the amino acid preparation.
Volume: 300mls with 33.75 g protein (Nitrogen is 5.4g so x 6.25)
– 1 g protein per 10 mls
—please note contains electrolytes too
Sodium 1 mmol per 10 mls
Potassium 0.8 mmol/ 10 mls
Magnesium 0.1 mmol/ 10mls
Calcium 0.07 mmol/ 10 mls
Phosphate 0.4 mmol/ 10 mls

3) Intralipid 20%. Volume: 255 mls.
1g fat per 5 mls

**Day of life**	**Total maintenance fluid volume for 24 hours**	**Volume of Vamin 18 novum**	**Volume of Intralipid**
1	60mls/kg	1g/kg protein =10 mls/kg	1g/kg fat = 5 mls/kg
2	80mls/kg	2g/kg protein =20 mls/kg	2g/kg fat =10 mls/kg
3	100mls/kg	3g/kg protein = 30mls/kg	3g/kg fat =15 mls/kg
4	120mls/kg	3g/kg protein = 30mls/kg	3g/kg fat =15 mls/kg
5 and onwards	*150 mls/kg*	*3g/kg protein = 30mls/kg*	*3g/kg fat = 15 mls/kg*

Maintenance fluids as per NICU protocol
Fluid volume adjusted for Prematurity as per NICU protocol

How to Calculate Parenteral Nutrition

1. Calculate the total maintenance fluid requirement for a 24 hour period.

For example, 2kg baby on day 5 of life = 150ml/kg/day = 300mls in 24 hours.

2. Calculate volume of Amino Acid - Vamin 18 according to weight as above.

For example 2kg baby on day 5: 2 (weight) x 30mls = 60mls.

3. Calculate volume of Intralipid according to weight as above.

For example 2kg baby day 5: 2 (weight) x 15mls = 30mls.

4. Take away the Vamin and Lipid volume from the total maintenance fluid requirement to obtain volume of Glucose

For example, for a 2kg baby on day 5: 300mls (total requirement) – 60 mls vamin - 30 mls lipid = 210mls Glucose

Electrolytes do not need to be added

5. Add the glucose and Vamin together and run over 24 hours

For example, for a 2kg baby on day 2: 210mls glucose plus 60mls vamin = 270 mls

270/24 = 11.25mls/hour.

6**. Do not mix lipid with anything else. Infuse over 24 hours**
Use a Y connector to run concurrently with vamin/glucose.

For example, for a 2kg baby on day 5: lipid is 30 mls/24 hours = 1.25 mls/hr

Use aseptic non touch technique throughout.

When re accessing PN chambers use surgical spirit liberally.

GASTROSCHISIS PATIENTS

Commence PN on day 1 of presentation or as soon as possible.

24 hours following closure of the gastroschisis defect, breastfeeding is to start as per the breastfeeding protocol.

When breastfeeding is established at 10 minutes 6 hourly, the PN can be reduced by half. When breastfeeding for 15 minutes 3 hourly, the PN can be stopped.

BREASTFEEDING PROTOCOL FOR GASTROSCHISIS STUDY

Kamuzu Central Hospital

PRIOR TO ARRIVAL AT KCH

From delivery to attendance at KCH - trophic breastfeeding (maximum 5 minutes per feed, 8 hourly) is allowed as part of kangaroo mother care. Alternatively baby may suckle on clean gauze soaked in breastmilk. Nasogastric (NG) tube is in situ and on free drainage.

WHEN SILO IS ON – give 1ml expressed breastmilk (EBM) 8 hourly orally via a syringe. EBM lollipops (EBM on gauze for baby to suck) can be used to sooth baby in between. Assist the mother with expressing regularly (every 2-3 hours) to maintain milk supply. NG tube is on free drainage with 4 hourly aspiration and ml for ml replacement of all losses with Ringer's lactate. Breastmilk volume is in addition to maintenance fluid requirements during this time.

FOLLOWING SILO REMOVAL & ABDOMINAL WALL CLOSURE

Day 0 - The day of closure, continue with NG on free drainage, 4 hourly aspiration and 1ml EBM 8 hourly.

Day 1 - Start breastfeeding 5 minutes, 8 hourly - keep NG on free drainage.

Day 2 - Clamp NG. Continue breastfeeding 5 minutes, 8 hourly.

(If unable to breastfeed, give 3mls/kg EBM, 8 hourly)

Day 3 - If tolerated increase breastfeeding to 5 minutes, 6 hourly.

(If unable to breastfeed, give 5mls/kg EBM, 6 hourly).

Day 4 - If tolerated increase breastfeeding to 10 minutes, 6 hourly. (Reduce Astymin by half = 18ml/kg/day)

(If unable to breastfeed, give 10mls/kg EBM, 6 hourly).

Day 5 - If tolerated increase breastfeeding to 10 minutes, 4 hourly.

(If unable to breastfeed, give 10mls/kg EBM, 4 hourly).

Day 6 - If tolerated increase breastfeeding to 15 minutes, 4 hourly. (Stop Astymin)

(If unable to breastfeed, give 12.5mls/kg EBM, 4 hourly).

Day 7 - If tolerated increase breastfeeding to 20 minutes, 2 hourly = full enteral feeding.

(If unable to breastfeed, give 12.5mls/kg EBM, 2 hourly).

*Reduce IV fluid maintenance volume by 20ml/kg/day as breastfeeding increases

NOTES

Bile stained aspirate or abdominal distension is not an indication to stop feed.

Single vomit – do not stop feed. Multiple vomits – revert to regime of previous day.

Nurse the neonate with their head/ chest slightly up in an inclined incubator or cot.

If the baby spikes a temperature/ becomes unwell - doctor to assess baby urgently.

Establishing breastfeeding will have some expected individual variance, but the aim is to establish breastfeeding as soon as possible to optimise chances of survival.
